# Pulmonary endothelial cell DNA methylation signature in pulmonary arterial hypertension

**DOI:** 10.18632/oncotarget.18031

**Published:** 2017-05-19

**Authors:** Aurélie Hautefort, Julie Chesné, Jens Preussner, Soni S Pullamsetti, Jorg Tost, Mario Looso, Fabrice Antigny, Barbara Girerd, Marianne Riou, Saadia Eddahibi, Jean-François Deleuze, Werner Seeger, Elie Fadel, Gerald Simonneau, David Montani, Marc Humbert, Frédéric Perros

**Affiliations:** ^1^ INSERM UMR_S 999, Hôpital Marie Lannelongue, Le Plessis Robinson, France; ^2^ Univ Paris-Sud, Faculté de Médecine, Université Paris-Saclay, Le Kremlin Bicêtre, France; ^3^ UMR_S 1087 CNRS UMR_6291, Institut du Thorax, Université de Nantes, CHU de Nantes, Centre National de Référence Mucoviscidose Nantes-Roscoff, Nantes, France; ^4^ Max-Planck-Institute for Heart and Lung Research, Member of the German Center for Lung Research (DZL), Bad Nauheim, Germany; ^5^ Centre National de Génotypage, CEA-Institut de Génomique, Evry, France; ^6^ AP-HP, Service de Pneumologie, Hôpital Bicêtre, Le Kremlin-Bicêtre, France; ^7^ INSERM U1046, Centre Hospitalier Universitaire Arnaud de Villeneuve, Montpellier, France; ^8^ Hôpital Marie Lannelongue, Service de Chirurgie Thoracique et Vasculaire, Le Plessis Robinson, France

**Keywords:** pulmonary arterial hypertension, epigenetic, DNA methylation, endothelial cells, ABC transporters

## Abstract

Pulmonary arterial hypertension (PAH) is a severe and incurable pulmonary vascular disease. One of the primary origins of PAH is pulmonary endothelial dysfunction leading to vasoconstriction, aberrant angiogenesis and smooth muscle cell proliferation, endothelial-to-mesenchymal transition, thrombosis and inflammation. Our objective was to study the epigenetic variations in pulmonary endothelial cells (PEC) through a specific pattern of DNA methylation.

DNA was extracted from cultured PEC from idiopathic PAH (*n* = 11), heritable PAH (*n* = 10) and controls (*n* = 18). DNA methylation was assessed using the Illumina HumanMethylation450 Assay. After normalization, samples and probes were clustered according to their methylation profile. Differential clusters were functionally analyzed using bioinformatics tools.

Unsupervised hierarchical clustering allowed the identification of two clusters of probes that discriminates controls and PAH patients. Among 147 differential methylated promoters, 46 promoters coding for proteins or miRNAs were related to lipid metabolism. Top 10 up and down-regulated genes were involved in lipid transport including ABCA1, ABCB4, ADIPOQ, miR-26A, BCL2L11. NextBio meta-analysis suggested a contribution of ABCA1 in PAH. We confirmed ABCA1 mRNA and protein downregulation specifically in PAH PEC by qPCR and immunohistochemistry and made the proof-of-concept in an experimental model of the disease that its targeting may offer novel therapeutic options.

In conclusion, DNA methylation analysis identifies a set of genes mainly involved in lipid transport pathway which could be relevant to PAH pathophysiology.

## INTRODUCTION

Pulmonary arterial hypertension (PAH) is a rare and severe condition defined by right heart catheterization as precapillary pulmonary hypertension (mean pulmonary arterial pressure ≥ 25 mmHg and pulmonary artery wedge pressure ≤ 15 mmHg), in the absence of other causes such as chronic thromboembolic pulmonary disease or chronic respiratory diseases and/or hypoxia [1]. PAH is the consequence of the progressive narrowing of the pulmonary precapillary vasculature that increases pulmonary vascular resistance. Pulmonary endothelial cell (PEC) dysfunction is a major player of PAH pathobiology. It is characterized by 1) PEC barrier breakdown, endothelial-to-mesenchymal transition and subsequent neointima formation [2], 2) pulmonary arterial vasoconstriction and remodeling through paracrine production of potent vasoconstrictors and growth factors that induce the contraction and the proliferation of underlying pulmonary arterial smooth muscle cells (PASMC) (medial hypertrophy) [3], pulmonary vascular inflammation [4], and *in situ* thrombosis [5]. Currently approved PAH therapies are all targeting three major PEC dysfunctional pathways involved in the abnormal PEC-PASMC crosstalk [6]. However none are curative, prompting the need of targeting other pathological signaling pathways in the future management of PAH.

We aimed at discovering genes, groups of co-regulated genes or pathways that would be differentially regulated in PAH and responsible for PEC dysfunction. Gene expression profiles are dependent on changes in the epigenome, including DNA methylation, histone modifications, and noncoding RNA regulation. DNA methylation is one of the most stable epigenetic modifications and traditionally regarded as the major mediator of epigenetic regulation. DNA methylation usually occurs at clusters of CpG dinucleotides called CpG islands, generally in promoter regions, often associated with the transcriptional inactivation of the affected gene [7]. DNA methylation contributes to the pathogenesis of pulmonary diseases, such as cancer, chronic obstructive pulmonary disease, and idiopathic pulmonary fibrosis [8, 9]. Two hypothesis-driven studies have already demonstrated methylation alterations in the genes coding for the superoxide dismutase 2 (SOD2) [10] and granulysin [11] in pulmonary hypertension (PH). However, studies examining DNA methylation on a global scale in PAH are lacking [12].

mRNA-based expression profile is the result of nutritional, growth factors, cytokines, neuro-hormonal stimulation present in the culture medium, and is also the result of the duration, the amplitude of these stimulations, and the combination of both at a certain time point of the life and of the cycle of the cell. Thus, mRNA-based expression profile is highly variable according to culture conditions. We hypothesized that stable and long-lasting DNA-based methylation profile represent the propensity for genes and groups of genes to be expressed or not, and that the sum of these propensities makes the ground for PEC dysfunction and PAH predisposition.

In this work, we provide for the first time a map of DNA methylation-based epigenetic predispositions to PAH, in isolated and cultured PEC. We have identified two signatures specific of PAH containing hypo-methylated and hyper-methylated promoters. A combination of bioinformatic tools was applied to determine the biological annotations of each pattern. We identified ABCA1 downregulation as one of the central hubs within the pathobiological networks responsible for PAH-related PEC dysfunction. We confirmed its mRNA and protein downregulation specifically in PAH PEC by qPCR and immunohistochemistry and made the proof-of-concept in experimental model of the disease that its targeting may offer novel therapeutic options.

## RESULTS

### Methylation profiling of PEC from PAH patients reveals two differential methylated signatures

**Figure 1:**
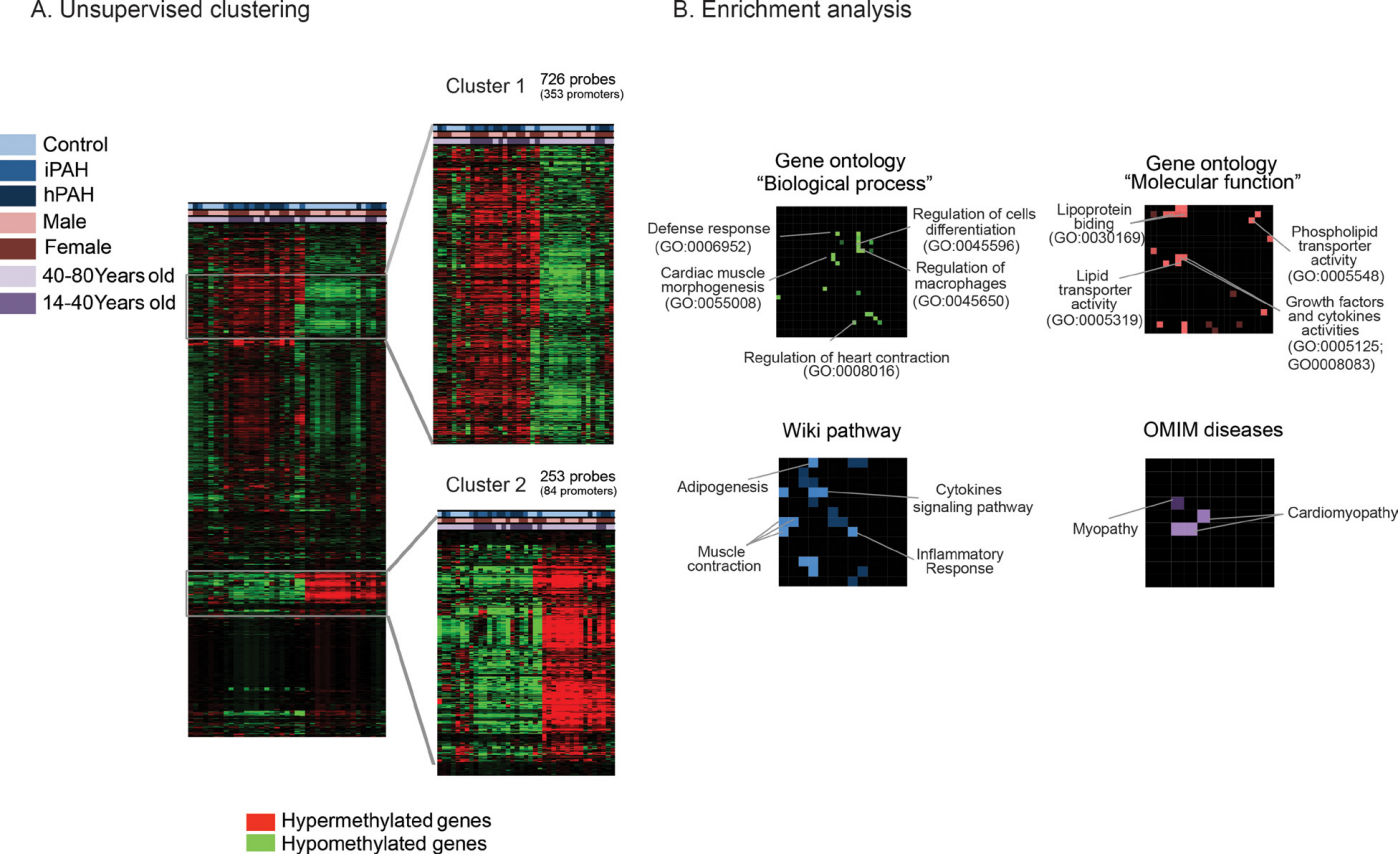
Differential DNA methylation pattern in pulmonary endothelial cells from patients with pulmonary arterial hypertension and controls (**A**) Heatmap representation of the differential methylated promoters coding for proteins and miRNAs in PAH patients (heritable + idiopathic) compared to controls. Two clusters (cluster 1 and cluster 2) were selected in function of the methylation levels (Over-methylated probes in red and under-methylated probes in green). (**B**) Enrichment analysis of the differentially methylated promoters as canvases (Network2Canvas software). For each canvas, brighter nodes represent lower *P*-value enriched terms.

**Figure 2:**
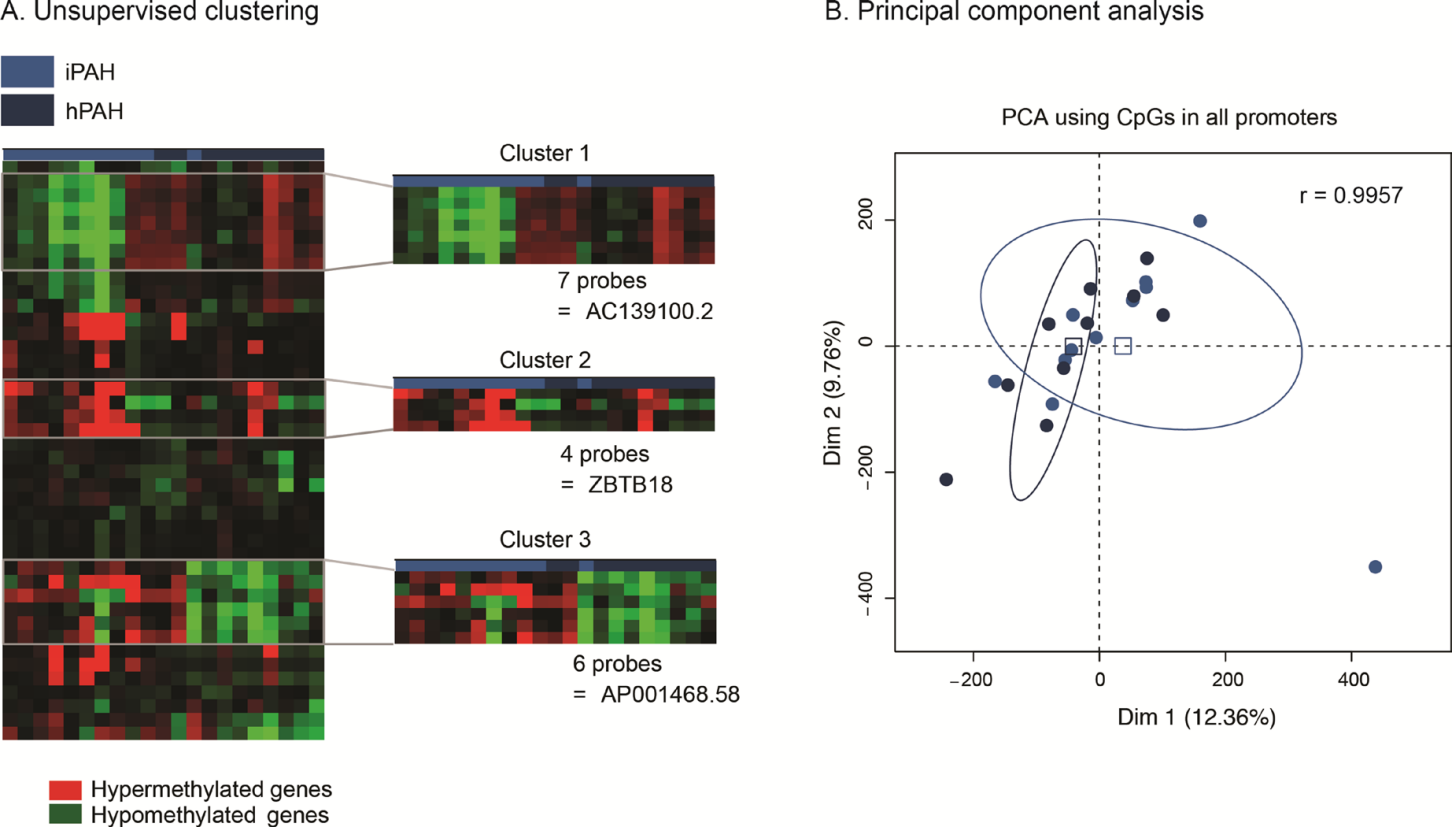
idiopathic and heritable PAH patients do not have a distinct endothelial methylation-based signature (**A**) Idiopathic PAH patients are represented in light blue and heritable PAH patients in dark blue. On the heatmap, the over- and under- expressed promoters are in red and green, respectively. Only three promoters displayed a differential methylation status, and only one is upstream of a known gene (*ZTB18*). (**B**) Principal Component Analysis (PCA) of the methylation profile (promoters) of idiopathic (*n* = 11) and heritable (*n* = 10) PAH patients. The Pearson correlation coefficient (r) was calculated comparing the CpG means across idiopathic and heritable groups.

DNA methylation assays were performed on cultured PEC from patients with PAH (11 idiopathic and 10 heritable) and 18 controls. Significant differentially methylated promoter regions were detected by ADMIRE and filtered using a FDR < 0.05 for each group (control versus idiopathic PAH; control vs heritable PAH and control vs idiopathic PAH and heritable PAH). We then used an unsupervised clustering approach to select signatures containing the most hyper- or hypo-methylated regions (Figure 1A). Among 4076 differentially methylated probes between control and PAH patients (idiopathic and heritable PAH), 726 (cluster 1) and 253 (cluster 2) probes discriminated both groups and corresponded to 352 hyper-methylated and 84 hypo-methylated promoters, respectively. Interestingly, only three promoters were differentially methylated between IPAH and HPAH patients suggesting that these patients present a similar methylation profile in their PEC despite different aetiologies (Figure 2A). We also generated a 2D plot (Principal Component Analysis (PCA) of the methylation profile (promoters) of IPAH and HPAH patients) to compare whole methylations between both populations, together with the calculation of Pearson's correlation coefficient. The PCA analysis confirmed that methylation pattern is similar between hPAH and iPAH (Figure 2B). The Pearson correlation values was calculated between the mean of all CpG sites in promoters from hPAH and from iPAH. The coefficient is 0,9957 which is high because the vast majority of the CpG sites do not change between hPAH and iPAH and those dominate the correlation. To rule out confounding factors, we additionally plotted the age and the gender showing no specific association to a cluster (Figure 1A).

Using N2C, we performed a functional analysis on a compiled list of hyper-methylated (cluster 1, *n* = 237) and hypo-methylated (cluster 2, *n* = 47) promoters coding for proteins (Figure 1B). This application allowed us to create canvases with enriched terms highlighted (*p*-value < 0.05) from 4 gene-set libraries (see method section). A part of the promoters discriminating PAH patients and controls is related to cardiomyopathy diseases with roles in cardiac muscle contraction and cardiac muscle morphogenesis (Figure 1B, Tables 1, 2, 3). Among them, promoters regulating DES (Desmin), ETS2 (ETS Proto-Oncogene 2, Transcription Factor), TBX1/5 (T-box 1/5), TERT (Telomerase Reverse Transcriptase) or MYH7 (Myosine β) were the most represented genes. In addition, we identified promoters involved in inflammatory process known to have a major role in PAH including ZAP70 (Syk-Related Tyrosine Kinase), STAT5A (Signal Transducer and Activator of Transcription 5A), COL1A1 (Alpha-1 Type I Collagen) and TNFSF4 (Tumor Necrosis Factor Superfamily Member 4) (Figure 1B, Tables 2 and 3). Interestingly, significant differential methylation was also found in gene promoters related to lipid transport activities such as ABCA1 (ATP-binding cassette 1), ADIPOQ (Adiponectin) and APOA4 (Apolipoprotein A4) (Figure 1B, Tables 2 and 3).

**Table 1:** Gene Ontology (GO) analysis related to “Biological process” of differentially methylated promoters in PAH patients

Term	*P*-value	Z-score	Genes
negative regulation of macrophage differentiation (GO:0045650)	0.000	−2.481	CRP;ABCA1;ADIPOQ;INHBA
negative regulation of cell differentiation (GO:0045596)	0.000	−2.214	CRP;ABCA1;ADIPOQ;TWIST2;INHBA;NKX2-5
negative regulation of myeloid cell differentiation (GO:0045638)	0.001	−2.446	CRP;ABCA1;ADIPOQ;INHBA
detection of abiotic stimulus (GO:0009582)	0.001	−2.109	RP1;OPN4;GNAT2;TCAP
defense response (GO:0006952)	0.001	−2.291	CX3CR1;CRP;APCS;NFAM1;STAB2;APOA4;INHBA;FAIM3;TYROBP;CD48;CD300C;S100A9;MBL2
negative regulation of foam cell differentiation (GO:0010745)	0.001	−1.782	CRP;ABCA1;ADIPOQ
heart development (GO:0007507)	0.002	−2.129	TBX1;TBX5;TDGF1;MYH7
cardiac muscle tissue morphogenesis (GO:0055008)	0.003	−2.008	TCAP;NKX2-5;MYH7
regulation of cell differentiation (GO:0045595)	0.003	−2.247	CRP;ABCA1;ZAP70;ADIPOQ;TWIST2;INHBA;TBX5;NKX2-5
phototransduction (GO:0007602)	0.004	−1.793	RP1;OPN4;GNAT2
anatomical structure morphogenesis (GO:0009653)	0.004	−2.148	COL1A1;TBX1;MAB21L1;TCAP;TBX5;NKX2-5;FGF1;TDGF1;FLI1;DKK3;MYH7
regulation of heart contraction (GO:0008016)	0.004	−1.872	DES;HSPB7;NKX2-5;MYH7
detection of light stimulus involved in visual perception (GO:0050908)	0.004	−1.727	RP1;OPN4;GNAT2
detection of visible light (GO:0009584)	0.005	−1.721	RP1;OPN4;GNAT2
regulation of myeloid cell differentiation (GO:0045637)	0.007	−1.904	CRP;ABCA1;ADIPOQ;INHBA
phospholipid efflux (GO:0033700)	0.011	−0.463	ABCA1;APOA4
cellular defense response (GO:0006968)	0.013	−1.765	CX3CR1;TYROBP;CD300C;FAIM3
antigen processing and presentation (GO:0019882)	0.013	−0.310	CLEC4M;CTSE
muscle contraction (GO:0006936)	0.017	−1.863	CHRNA1;DES;MYOT;GJA5;TCAP
protein-lipid complex assembly (GO:0065005)	0.018	−0.948	ABCA1;APOA4
negative regulation of lipid storage (GO:0010888)	0.018	−0.890	CRP;ABCA1
response to external stimulus (GO:0009605)	0.020	−2.123	CX3CR1;CRP;APCS;RP1;OPN4;GNAT2;NFAM1;TCAP;INHBA;S100A9;MBL2
phospholipid transport (GO:0015914)	0.021	−1.010	ABCA1;APOA4
acute-phase response (GO:0006953)	0.021	−0.916	APCS;MBL2
reverse cholesterol transport (GO:0043691)	0.021	−0.911	ABCA1;APOA4
multicellular organismal metabolic process (GO:0044236)	0.021	−0.871	COL1A1;APOA4
cell-cell junction assembly (GO:0007043)	0.023	−0.792	GJD3;GJA5
detection of stimulus (GO:0051606)	0.025	−1.572	RP1;OPN4;GNAT2;TCAP
cholesterol efflux (GO:0033344)	0.029	−1.103	ABCA1;APOA4
positive regulation of cell differentiation (GO:0045597)	0.032	−1.745	ZAP70;INHBA;TBX5;NKX2-5
regulation of gene-specific transcription (GO:0032583)	0.032	−1.660	GFI1;TNFSF4;TBX5;GATA2
regulation of cholesterol transport (GO:0032374)	0.036	−1.208	ADIPOQ;APOA4
regulation of specific transcription from RNA polymerase II promoter (GO:0010551)	0.037	−1.477	GFI1;TBX5;GATA2
actin filament-based process (GO:0030029)	0.039	−1.642	BMP10;ARHGDIB;TCAP;KPTN;MYH7
negative regulation of gene-specific transcription (GO:0032582)	0.042	−1.063	GFI1;TNFSF4
leukocyte adhesion (GO:0007159)	0.042	−0.950	CLEC4M;APOA4
regulation of transcription (GO:0045449)	0.048	−1.857	TBX1;TFAP2A;BMP10;GFI1;NFAM1;NR1I2;ARID5A;INHBA;GDF6;TBX5;GATA2;ETS2;SOX18;TNFSF4;ID3;ALX1;SLC2A4RG;TEAD3
cell-cell junction organization (GO:0045216)	0.050	−1.099	GJD3;GJA5
enzyme linked receptor protein signaling pathway (GO:0007167)	0.050	−1.676	BMP10;NRTN;PTN;GDF6;FGF1;TDGF1

Only GO categories with *P*-value less than 5% are represented.

**Table 2:** Gene Ontology (GO) analysis related to “molecular activity” of differentially methylated promoters in PAH patients

Term	*P*-value	Z-score	Genes
RNA polymerase II regulatory region DNA binding (GO:0001012)	0.00	−2.42	STAT5A;TFAP2A;NR1I2;TBX5;GATA2;ACTB;FLI1;ETS2;NR2F6;SOX18;ELF5;HAND2;ALX1;NKX2-5
transcription regulatory region sequence-specific DNA binding (GO:0000976)	0.00	−2.42	STAT5A;TFAP2A;NR1I2;TBX5;GATA2;ACTB;FLI1;ETS2;NR2F6;SOX18;ELF5;HAND2;ALX1;NKX2-5
cytidine deaminase activity (GO:0004126)	0.00	−2.03	APOBEC1;APOBEC2;AICDA
virion binding (GO:0046790)	0.00	−2.27	CRP;APCS;CLEC4M
RNA polymerase II transcription regulatory region sequence-specific DNA binding transcription factor activity involved in positive regulation of transcription (GO:0001228)	0.00	−2.28	TFAP2A;SOX18;ELF5;HAND2;NR1I2;ALX1;TBX5;GATA2;NKX2-5;FLI1
deaminase activity (GO:0019239)	0.00	−2.61	APOBEC1;APOBEC2;AMPD2;AICDA
RNA polymerase II distal enhancer sequence-specific DNA binding (GO:0000980)	0.00	−2.23	TFAP2A;GATA2;ACTB;FLI1;NR2F6
hydrolase activity, acting on carbon-nitrogen (but not peptide) bonds, in cyclic amidines (GO:0016814)	0.00	−2.36	APOBEC1;APOBEC2;AMPD2;AICDA
growth factor activity (GO:0008083)	0.00	−2.29	BMP10;CLEC11A;NRTN;PTN;INHBA;GDF6;FGF1;TDGF1
enhancer sequence-specific DNA binding (GO:0001158)	0.00	−2.22	TFAP2A;GATA2;ACTB;FLI1;NR2F6
sequence-specific DNA binding RNA polymerase II transcription factor activity (GO:0000981)	0.00	−2.30	TFAP2A;GFI1;NR1I2;TBX5;GATA2;FLI1;ETS2;NR2F6;SOX18;ELF5;HAND2;ALX1;NKX2-5
enhancer binding (GO:0035326)	0.01	−2.20	TFAP2A;GATA2;ACTB;FLI1;NR2F6
hormone activity (GO:0005179)	0.01	−2.22	BMP10;ADIPOQ;OXT;INHBA;CGA;METRNL
RNA polymerase II core promoter proximal region sequence-specific DNA binding transcription factor activity (GO:0000982)	0.01	−2.21	TFAP2A;SOX18;GFI1;HAND2;GATA2;NKX2-5;FLI1;ETS2
calcium activated cation channel activity (GO:0005227)	0.01	−2.33	CATSPER4;TRPM5;KCNK18
cytokine activity (GO:0005125)	0.01	−2.25	BMP10;TNFSF4;CMTM5;ADIPOQ;XCL2;SCGB3A1;INHBA;GDF6
quaternary ammonium group binding (GO:0050997)	0.01	−2.22	CRP;CHRNA1;APOA4
receptor serine/threonine kinase binding (GO:0033612)	0.01	−1.28	BMP10;INHBA
opsonin binding (GO:0001846)	0.01	−1.14	CRP;APCS
cholesterol transporter activity (GO:0017127)	0.01	−1.06	ABCA1;APOA4
olfactory receptor activity (GO:0004984)	0.01	−2.09	OR10J1;OR51B2;OR10J5;OR2F1;OR9A2;OR51B6;OR51B5;OR52B4;OR10G7;OR7D4;OR2A5;OR2A2
sterol transporter activity (GO:0015248)	0.01	−1.20	ABCA1;APOA4
passive transmembrane transporter activity (GO:0022803)	0.01	−2.25	CHRNA1;GJD3;ASIC5;TRPV6;CATSPER4;GABRA5;GJA5;PDPN;ORAI1;TRPM5;KCNK18;KCNK4
channel activity (GO:0015267)	0.01	−2.23	CHRNA1;GJD3;ASIC5;TRPV6;CATSPER4;GABRA5;GJA5;PDPN;ORAI1;TRPM5;KCNK18;KCNK4
protein homodimerization activity (GO:0042803)	0.02	−2.13	CRP;TBX1;TFAP2A;CADM3;ADIPOQ;FAM109B;APOA4;GDF6;PRLR;TERT;HAND2;CHMP4C;CTSE;NKX2-5;UGT1A8;S100A10
antioxidant activity (GO:0016209)	0.02	−1.87	TP53INP1;APOA4;LTC4S;S100A9
aspartic-type endopeptidase activity (GO:0004190)	0.02	−1.85	PIP;CTSE;ASPRV1
RNA polymerase II core promoter proximal region sequence-specific DNA binding transcription factor activity involved in positive regulation of transcription (GO:0001077)	0.02	−1.91	TFAP2A;SOX18;HAND2;GATA2;NKX2-5;FLI1
ion gated channel activity (GO:0022839)	0.02	−1.86	CATSPER4;TRPM5;KCNK18
aspartic-type peptidase activity (GO:0070001)	0.02	−1.76	PIP;CTSE;ASPRV1
enzyme activator activity (GO:0008047)	0.02	−2.15	DAOA;PCOLCE2;TBC1D21;NCF4;ARHGDIB;PLXNB2;ARHGEF10L;APOA4;PTPLAD1;LTC4S;RASAL3;PRLR
structural constituent of muscle (GO:0008307)	0.02	−1.71	NEBL;MYOT;TCAP
phospholipid transporter activity (GO:0005548)	0.02	−1.68	ABCA1;PITPNM3;PLSCR4
drug transmembrane transporter activity (GO:0015238)	0.02	−1.11	SLC47A1;ABCB4
substrate-specific channel activity (GO:0022838)	0.02	−2.03	CHRNA1;GJD3;ASIC5;TRPV6;CATSPER4;GABRA5;PDPN;ORAI1;TRPM5;KCNK18;KCNK4
scavenger receptor activity (GO:0005044)	0.03	−1.55	TMPRSS5;STAB2;HHIPL1
RNA polymerase II core promoter proximal region sequence-specific DNA binding (GO:0000978)	0.03	−1.80	TFAP2A;SOX18;NKX2-5;ACTB;FLI1;ETS2
lipid transporter activity (GO:0005319)	0.03	−1.68	ABCA1;PITPNM3;PLSCR4;APOA4

Only GO categories with P-value less than 5% are represented.

**Table 3:** Enrichment analysis based on the wiki pathway and OMIM disease libraries for differentially methylated promoters in PAH patients

Term in each category	*P*-value	Z-score	Genes
Wiki pathway			
Hs_Myometrial_Relaxation_and_Contraction_Pathways_WP289_21289	0.002	−1.848	IGFBP1;GPR182;ADCY4;GNG8;OXT;ACTB;ETS2
Hs_Statin_Pathway_PharmGKB_WP430_29996	0.025	−1.430	ABCA1;APOA4
Hs_Statin_Pathway_PharmGKB_WP430_21586	0.025	−1.219	ABCA1;APOA4
Hs_Nuclear_receptors_in_lipid_metabolism_and_toxicity_WP299_21309	0.044	−1.191	ABCA1;ABCB4
Hs_Inflammatory_Response_Pathway_WP453_21632	0.050	−1.081	COL1A1;ZAP70
Hs_Adipogenesis_WP236_28133	0.054	−1.517	STAT5A;ADIPOQ;ID3;GATA2
Hs_T_Cell_Receptor_Signaling_Pathway_WP69_20823	0.063	−1.540	STAT5A;ZAP70;NFAM1;ARHGDIB
Hs_Striated_Muscle_Contraction_WP383_21484	0.073	−0.866	DES;TCAP
Hs_IL-7_Signaling_Pathway_WP205_21111	0.088	−1.116	STAT5A;BCL2L11
Hs_IL-3_Signaling_Pathway_WP286_21281	0.108	−1.278	STAT5A;BCL2L11;GATA2
OMIM Disease			
cardiomyopathy,_dilated	0.000	−1.973	DES;TCAP;LDB3;MYH7
cardiomyopathy	0.001	−1.861	DES;TCAP;LDB3;MYH7
myopathy	0.011	−1.456	DES;LDB3;MYH7
fibrosis	0.124	0.313	TERT

Only enriched terms with *P*-value less than 5% are represented.

### PEC from PAH patients exhibit major epigenetic alterations in the metabolic pathway

**Figure 3:**
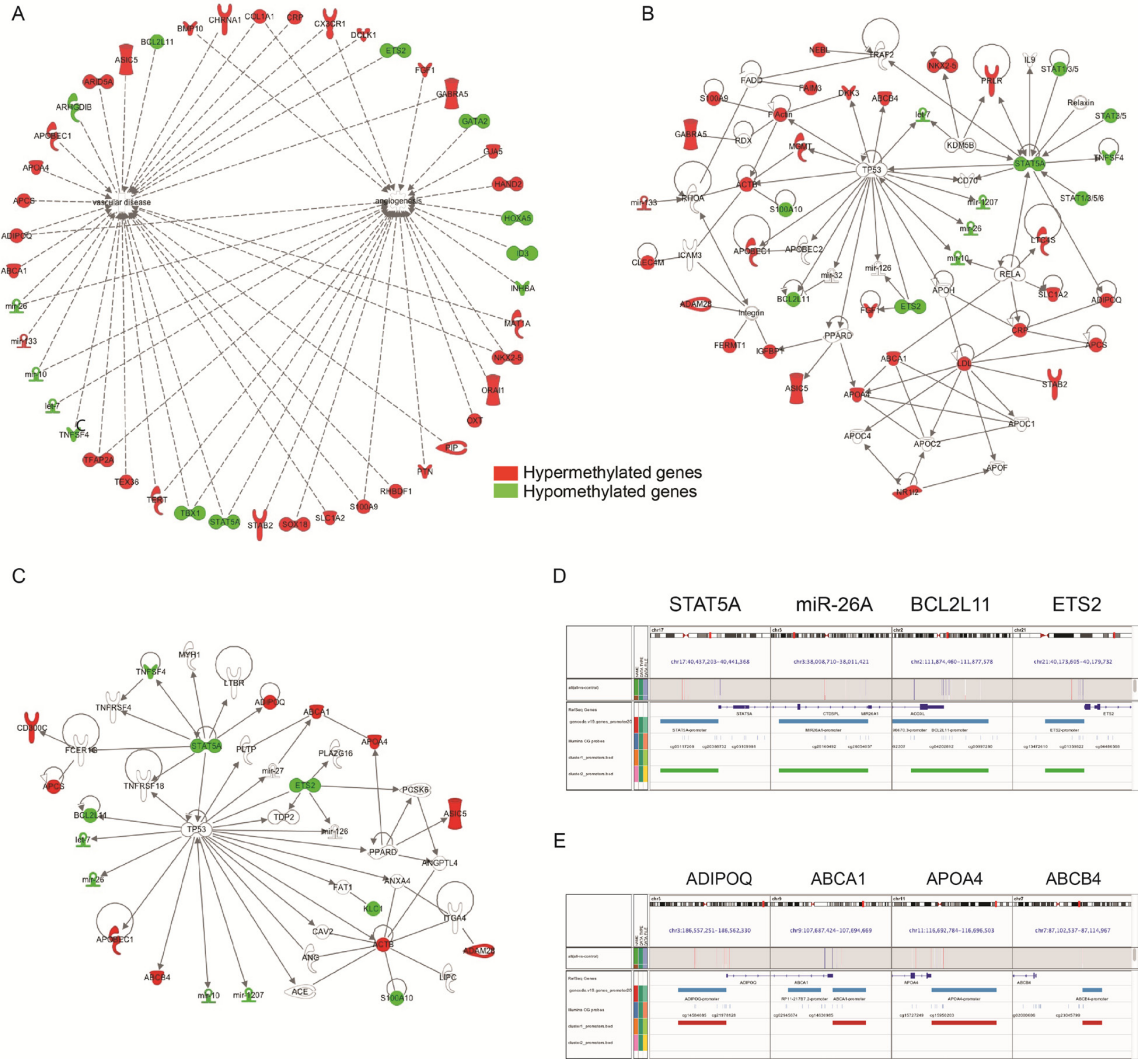
Epigenetic variations in promoters related to the lipid and cholesterol pathway (**A**–**C**) Gene network generated by Ingenuity Pathway Analysis (IPA) on the differentially methylated promoters between PAH patients and controls. Over-methylated genes are represented in red and under-methylated genes in green. (**D**–**E**) Visualization of the under-methylated (D) versus hyper-methylated locus (E) in a selection of promoters with Integrative Genomics Viewer (IGV).

To visualize and understand potential functional interactions of the identified genes, we submitted the same gene lists to Ingenuity pathway analysis (IPA). IPA results in dense networks relating genes to distinct biological functions, as shown in Figure 3A–3C. Figure 3A shows all hyper-methylated (in red) and hypo-methylated (in green) genes potentially correlated with vascular diseases and angiogenesis. DNA hyper-methylation has been observed in promoters already described as deregulated in PAH pathogenesis including the fibroblast growth factor (FGF) family, the receptor of the inflammatory chemokine fractalkine (CX3CR1) and the tumor necrosis factor family (TNFSF4, alias OX40L) [13]. Importantly, we further confirmed the results from unsupervised clustering (Figure 1A) with the presence of a set of interconnected genes related to lipid metabolism such as ADIPOQ, APOA4 and ABCA1 (Figure 3A).

Based on these observations, we conducted a second pathway analysis from the full list of differentially methylated metabolic genes given by IPA (Table 4). Among 48 genes, we found that 28 hyper-methylated and 11 hypo-methylated genes were directly connected to the same network “cholesterol and lipid transport” (Figure 3B). When we focused on the ten top candidates genes hyper- or hypo-methylated (Figure 3C), TP53 (tumor protein 53) was presented directly or indirectly as a common denominator of all genes. In addition, this network integrates other genes with a proved activity in PAH such as angiotensin converting enzyme (ACE), angiotensin (ANG) or PPAR-δ [14]. Among these top genes, we selected the most differentially methylated promoter regions across the visualization of the targeted loci (Figure 3D and 3E). Altogether, these data suggest that cholesterol metabolism is closely associated with PAH.

**Table 4:** List of the 48 differentially methylated promoter genes identified by Ingenuity pathway analysis (IPA) to be involved in metabolic processes

FC	Symbol	Gene Name	Location	Type(s)
2,000	ABCA1	ATP-binding cassette, sub-family A (ABC1), member 1	Plasma Membrane	transporter
2,000	ABCB4	ATP-binding cassette, sub-family B (MDR/TAP), member 4	Plasma Membrane	transporter
2,000	ACTB	actin, beta	Cytoplasm	other
−2,000	ADAM12	ADAM metallopeptidase domain 12	Plasma Membrane	peptidase
2,000	ADIPOQ	adiponectin, C1Q and collagen domain containing	Extracellular Space	other
2,000	APCS	amyloid P component, serum	Extracellular Space	other
2,000	APOA4	apolipoprotein A-IV	Extracellular Space	transporter
2,000	APOBEC1	apolipoprotein B mRNA editing enzyme, catalytic polypeptide 1	Cytoplasm	enzyme
2,000	ASIC5	acid sensing (proton gated) ion channel family member 5	Plasma Membrane	ion channel
−2,000	BCL2L11	BCL2-like 11 (apoptosis facilitator)	Cytoplasm	other
2,000	CD300C	CD300c molecule	Plasma Membrane	transmembrane receptor
2,000	CDH17	cadherin 17, LI cadherin (liver-intestine)	Plasma Membrane	transporter
2,000	CLEC4M	C-type lectin domain family 4, member M	Plasma Membrane	other
2,000	COL1A1	collagen, type I, alpha 1	Extracellular Space	other
2,000	CRP	C-reactive protein, pentraxin-related	Extracellular Space	other
2,000	CX3CR1	chemokine (C-X3-C motif) receptor 1	Plasma Membrane	G-protein coupled receptor
2,000	DKK3	dickkopf WNT signaling pathway inhibitor 3	Extracellular Space	cytokine
−2,000	ETS2	v-ets avian erythroblastosis virus E26 oncogene homolog 2	Nucleus	transcription regulator
2,000	FAIM3	Fas apoptotic inhibitory molecule 3	Plasma Membrane	other
2,000	FERMT1	fermitin family member 1	Plasma Membrane	other
2,000	FGF1	fibroblast growth factor 1 (acidic)	Extracellular Space	growth factor
2,000	GABRA5	gamma-aminobutyric acid (GABA) A receptor, alpha 5	Plasma Membrane	ion channel
2,000	HBG2	hemoglobin, gamma G	Cytoplasm	other
2,000	IGFBP1	insulin-like growth factor binding protein 1	Extracellular Space	other
−2,000	KLC1	kinesin light chain 1	Cytoplasm	other
−2,000	let-7	microRNA let-7a-1	Cytoplasm	microRNA
2,000	LPPR4	lipid phosphate phosphatase-related protein type 4	Plasma Membrane	phosphatase
2,000	LTC4S	leukotriene C4 synthase	Cytoplasm	enzyme
2,000	MAT1A	methionine adenosyltransferase I, alpha	Cytoplasm	enzyme
2,000	MGMT	O-6-methylguanine-DNA methyltransferase	Nucleus	enzyme
−2,000	mir-10	microRNA 100	Other	microRNA
2,000	mir-133	microRNA 133b	Cytoplasm	microRNA
−2,000	mir-26	microRNA 26b	Cytoplasm	microRNA
2,000	MT2A	metallothionein 2A	Cytoplasm	other
2,000	NEBL	nebulette	Plasma Membrane	other
2,000	NKX2-5	NK2 homeobox 5	Nucleus	transcription regulator
2,000	NR1I2	nuclear receptor subfamily 1, group I, member 2	Nucleus	ligand-dependent nuclear receptor
2,000	PRLR	prolactin receptor	Plasma Membrane	transmembrane receptor
2,000	RNLS	renalase, FAD-dependent amine oxidase	Extracellular Space	enzyme
−2,000	S100A10	S100 calcium binding protein A10	Cytoplasm	other
2,000	S100A9	S100 calcium binding protein A9	Cytoplasm	other
2,000	SLC1A2	solute carrier family 1 (glial high affinity glutamate transporter), member 2	Plasma Membrane	transporter
2,000	STAB2	stabilin 2	Plasma Membrane	transmembrane receptor
−2,000	TNFSF4	tumor necrosis factor (ligand) superfamily, member 4	Extracellular Space	cytokine
2,000	TYROBP	TYRO protein tyrosine kinase binding protein	Plasma Membrane	transmembrane receptor
2,000	ADAM28	ADAM Metallopeptidase Domain 28	Extracellular Space	enzyme
−2,000	STAT5A	Signal Transducer And Activator Of Transcription 5A	Nucleus	transcription factor
−2,000	MIR1207	MicroRNA 1207	Cytoplasm	microRNA

FC = Fold change (2.000 for hyper-methylated genes and −2.000 for hypomethylated genes).

### ABCA1, a target in the pathogenesis of PAH

**Figure 4:**
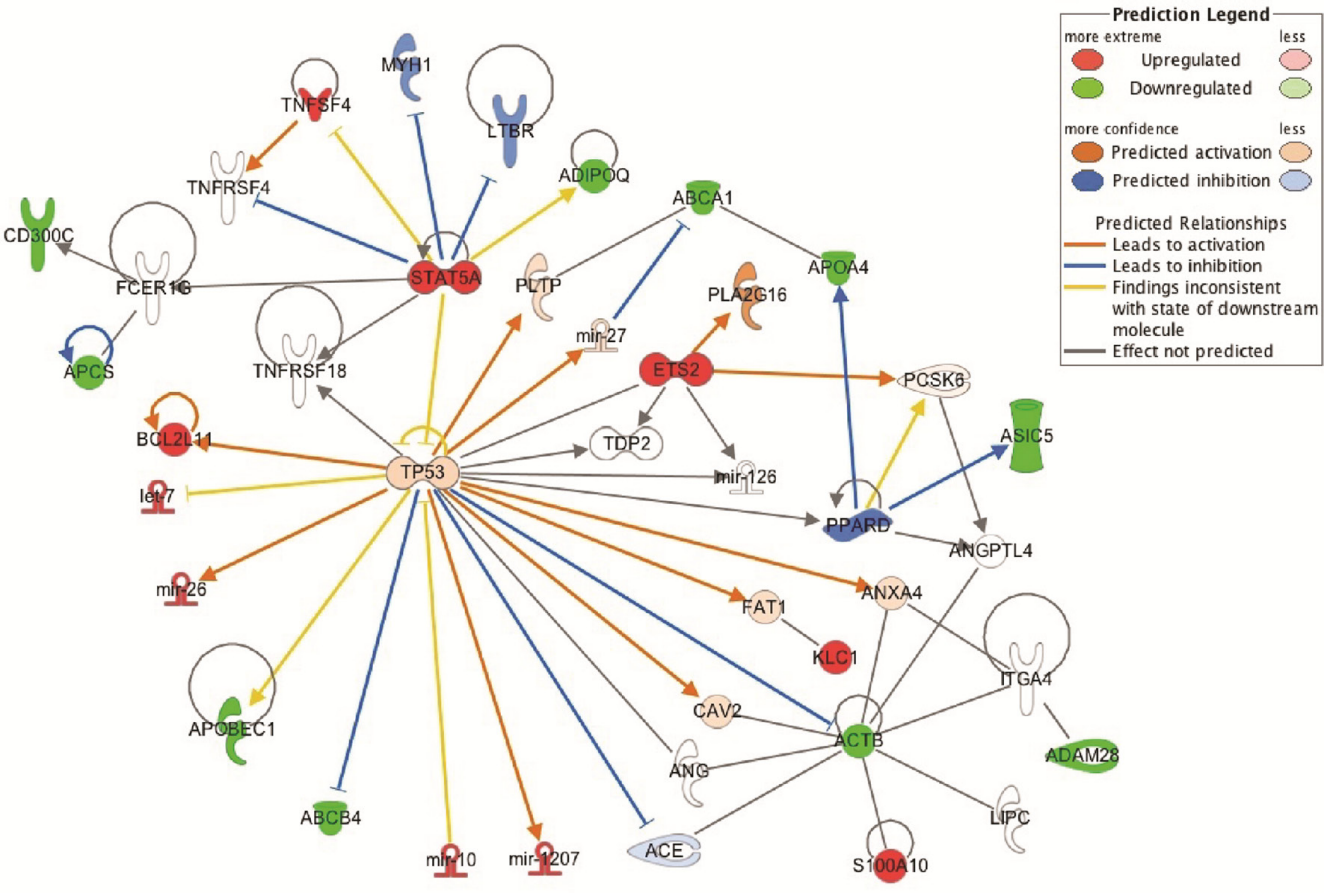
Prediction analysis by IPA on the expression of genes related to the lipid pathway In red are represented the over-expressed genes (= hypomethylated promoters) and in green the under-expressed genes (= hypermethylated promoters).

**Figure 5:**
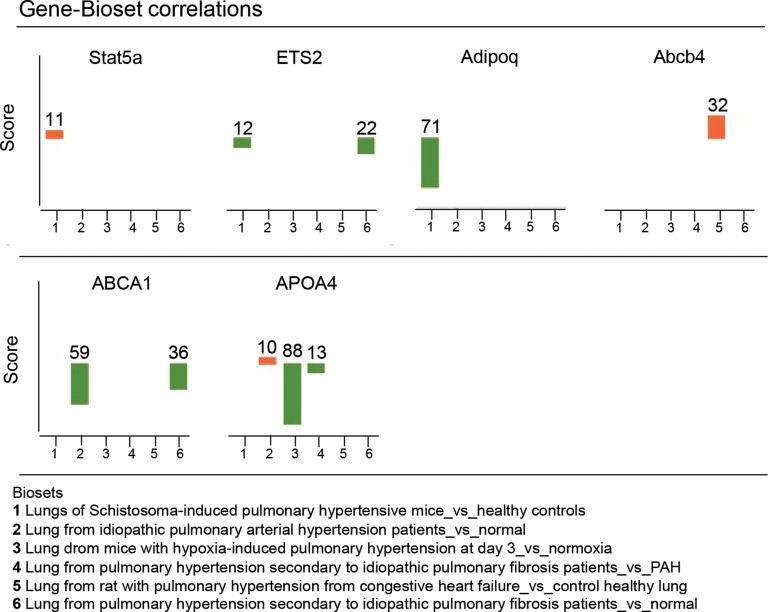
Validation of the DNA methylation profile using public gene expression data sets obtained in lung tissues from experimental and human pulmonary hypertension Based on the 10 top genes displaying a significantly different methylation profile described in Figure 3E, a meta-analysis was performed using the NextBio platform. A pair-wise correlation score was computed between 6 biosets (or studies) related to PAH. According to a disease or tissue, this score determines the highly relevant genes through the normalized rank of the gene in the bioset, the number of biosets containing the gene and the number of studies. In red are represented the over-expressed genes (positive fold-change) and in green the under-expressed genes (negative fold-change).

**Figure 6:**
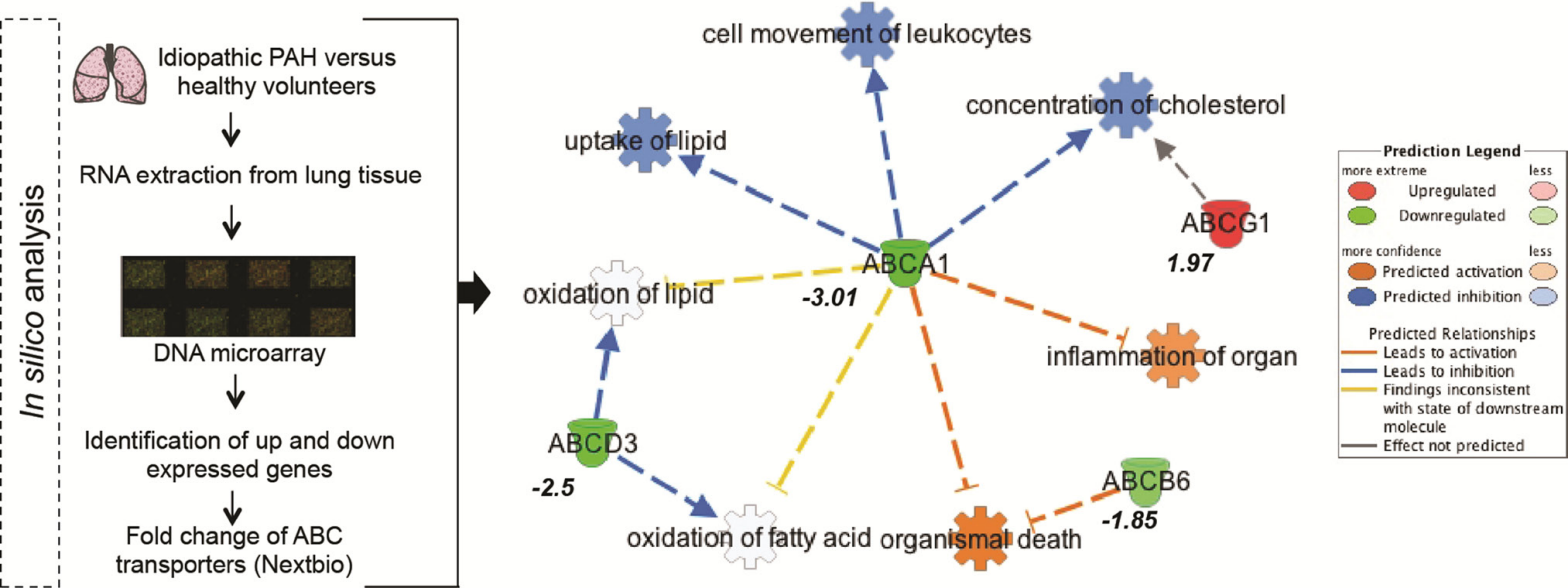
In silico gene expression analysis reveals a major functional role for ABCA1 Given a list of 14 genes coding for the ABC transporters, Nextbio provided the Fold-change for each gene related to 1 bioset [86]. Thanks to Ingenuity Pathway Analysis (IPA), a network was constructed to measure the potential impact (or prediction) of each ABC transporter on «diseases or function annotations». In red are represented the over-expressed genes and in green the under-expressed genes.

**Figure 7:**
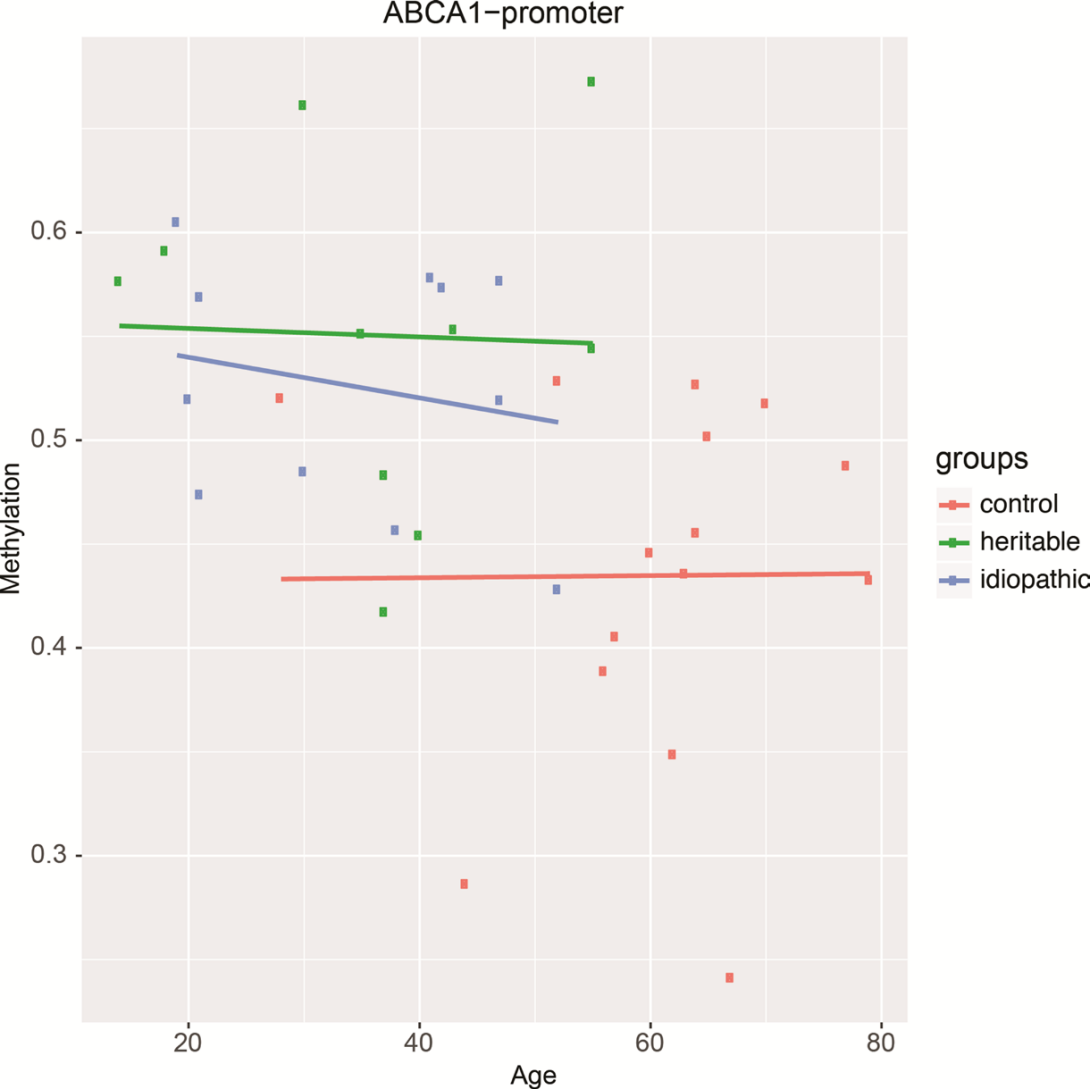
The methylation status of the ABCA1 promoter is not related to age in PEC from controls, and idiopathic and heritable PAH

**Figure 8:**
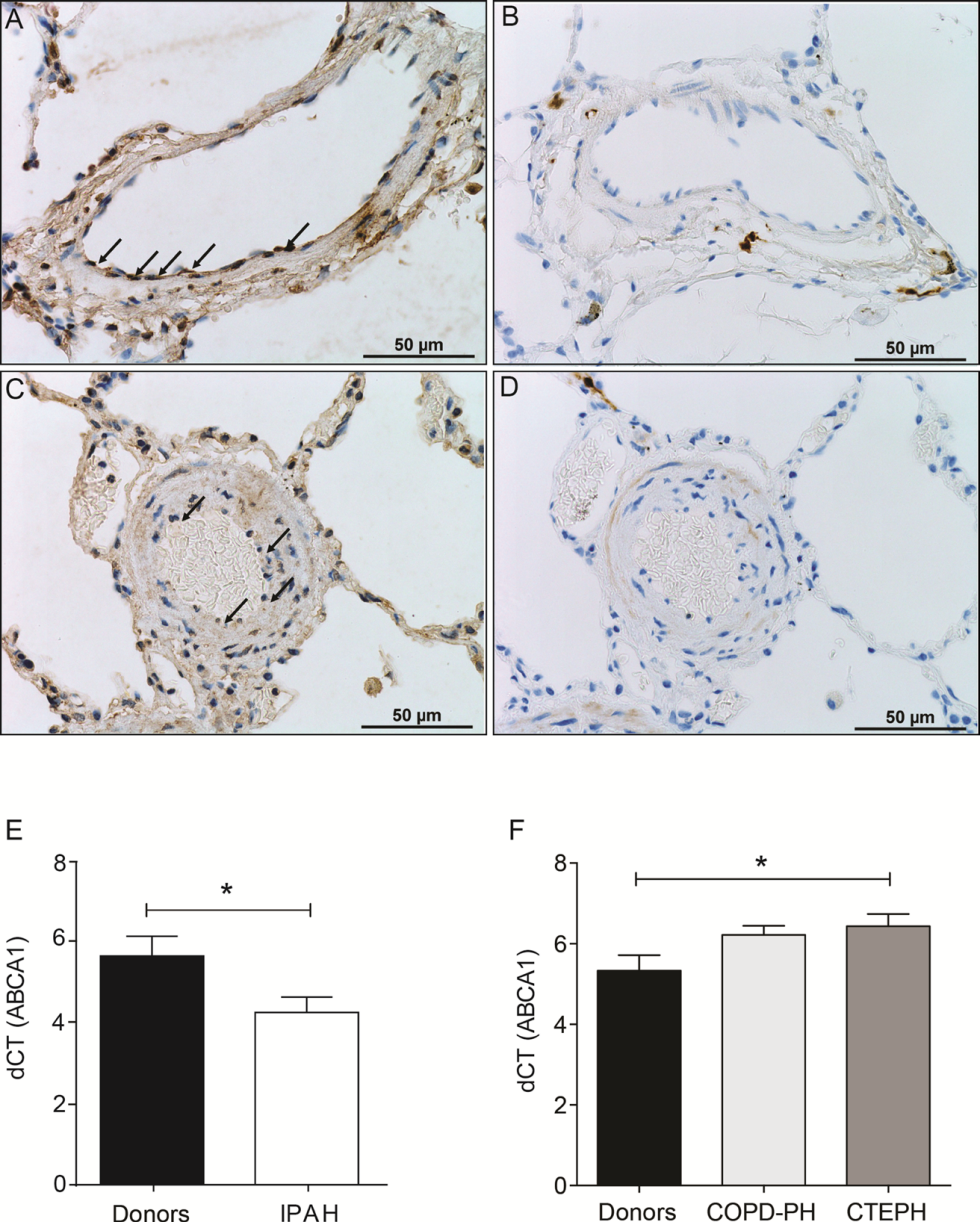
Loss of protein expression of ABCA1 in PAH PEC and decreased mRNA expression of ABCA1 in lungs from PAH patients Immunohistochemistry with mouse anti-ABCA1 antibody (**A** and **C**) or control isotype (IgG2b) mouse antibody (**B** and **D**) in healthy (A and B) or PAH (C and D) lungs. Real-time polymerase chain reaction quantification of gene expression showed a significant decrease of ABCA1 mRNA normalized to b-actin mRNA in total lungs from patients with IPAH (*n* = 7) compared with control subjects (donors) (*n* = 7) (**E**). There was no ABCA1 down regulation in CTEPH (*n* = 8) and COPD-PH (*n* = 9) compared with control subjects (donors) (*n* = 7) (**F**). Data are represented as mean +/ SEM, *P < 0.05.

**Figure 9:**
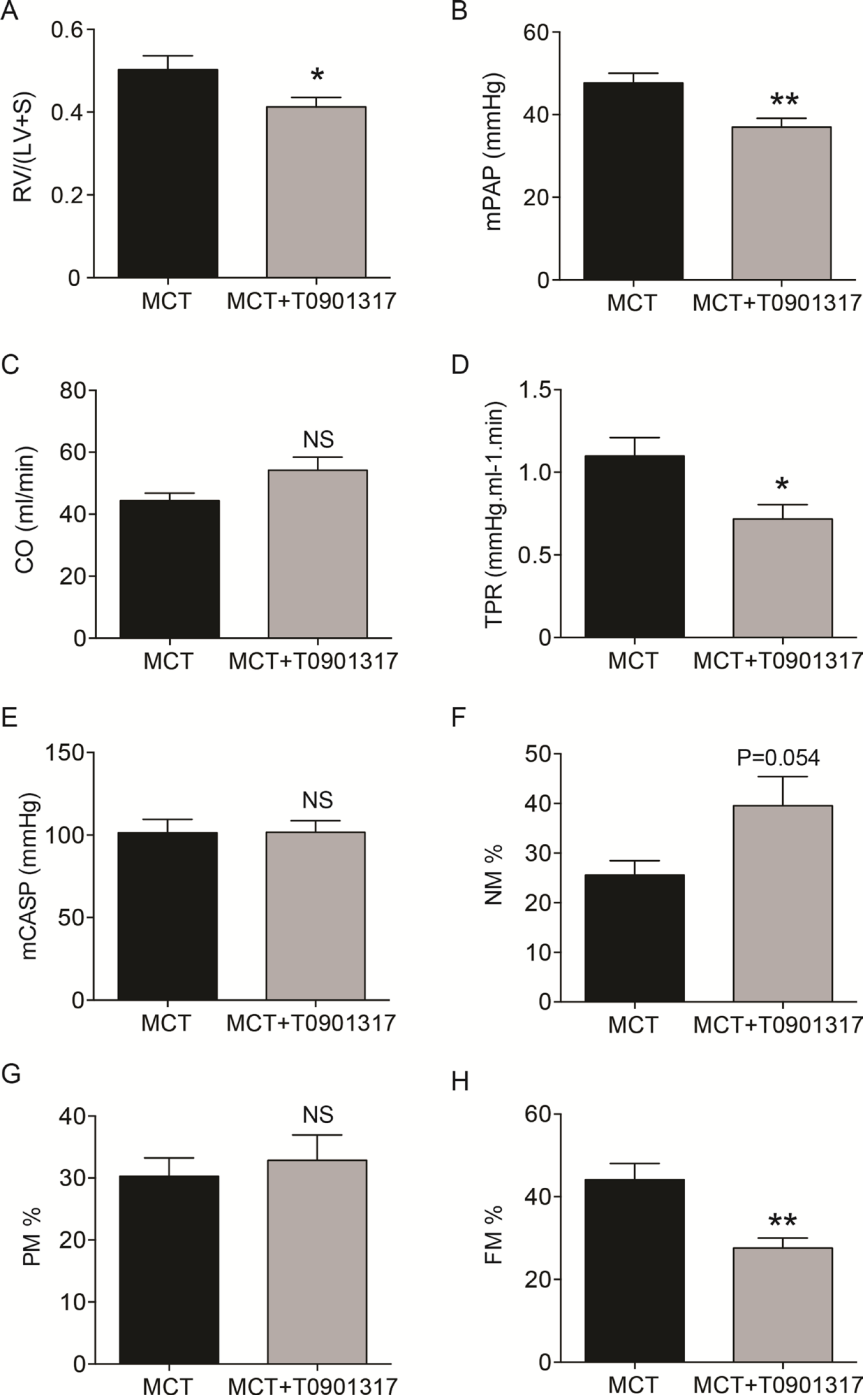
*In vivo* effects of the ABCA1-activating compound T0901317 on hemodynamics, right heart hypertrophy and pulmonary vascular remodeling in monocrotaline-induced pulmonary hypertension in rats Two groups were compared: 1) a monocrotaline (MCT) and solvent (ethanol)-exposed group (MCT); 2) a MCT-exposed and 10 mg/kg/day (days 14–21) T0901317-treated group (MCT+T0901317). *In vivo* effects of T0901317 on (**A**) Fulton's index of right ventricular hypertrophy, calculated as the ratio of the right ventricular weight–to–left ventricular plus septal weight (RV/LV+S), (**B**) mean pulmonary artery pressure (mPAP) (mm Hg), (**C**) cardiac output (CO) (ml/min), (**D**) total pulmonary vascular resistance (TPR) (mmHg.ml^−1^.min) and (**E**) mean carotid artery systemic pressure (mCASP) (mmHg). The analysis of the neomuscularization of normally non-muscularized small distal pulmonary artery (PA) (≤ 50 μm) is a common and robust way to quantify the degree of remodeling of the rat pulmonary vasculature. (**F**) Percentage of non-muscularized (NM) PA. (**G**) Percentage of partially muscularized (PM) PA. (**H**) Percentage of fully muscularized (FM) PA. MCT and MCT+ T0901317: 5 ≤ n ≤ 7. Numbers vary due to death of the animals and/or failure to record the pressures. *P < 0.05, **P < 0.01.

In most cases, methylation of CpG dinucleotides leads to an altered expression of the gene under regulatory control. By assuming that DNA methylation of the genes identified by IPA was negatively correlated with their gene expression level, we simulated a predictive relationship between each genes included in the network “metabolism” (Figure 3C, Figure 4). Interestingly, TP53 may activate BCL2L11, ETS2, miR-26 and inhibit the expression of ABCB4 or ABCA1. To validate this potential regulatory network, we conducted a meta-analysis on the same genes using Nextbio on six previously published studies (biosets) performed on PAH lung from animal or human (Figure 5). Out of 8 genes (Figure 3D and 3E), only 6 genes were already described in lungs of PAH patients (Figure 5). Among them, only ABCA1, ADIPOQ and APOA4 were correlated with their methylation status. Indeed, they were found under-expressed (in green) in one (for ADIPOQ) or two biosets (for ABCA1, ADIPOQ) with a score comprised between 10 and 88 (Figure 5). Although the role of ADIPOQ was already identified in PAH [15], the implication of ABCA1 remains unexplored. The ABCA1 gene is a member of the superfamily of ATP Binding cassette (ABC) transporters involved in the regulation of cholesterol and phospholipid homeostasis. To date, 46 ABC transporters have been identified as important mediators in human diseases. Interestingly, when we conduct a second meta-analysis on the 46 genes coding for ABC transporters, we showed that 14 of them were under-expressed particularly in lung from idiopathic PAH patients compared to controls (Table 5). Interestingly, ABCA1 was the candidate gene with the highest fold change, highlighting the importance of its functional activity (FC = −3.01, Figure 6). Indeed, an altered regulation of ABCA1 can lead to an increase of inflammation and apoptosis, two majors contributing factors to PAH (Figure 6) but also can impair the cholesterol and lipid transport and leukocytes migration. Since transplanted PAH patients are younger than lung cancer-affected controls, it was impossible to match both groups for age. It is important to note that ABCA1 methylation status was not correlated to the age (Figure 7). Additionally, we determined by immunohistochemistry that there is a specific *in situ* loss of ABCA1 expression in pulmonary artery endothelial cells from PAH patients (Figure 8A–8D). We confirmed by qPCR the downregulation of ABCA1 at the mRNA level in PAH lungs as compared to age- and sex-matched unused transplant donors obtained from University Hospital Giessen (Giessen, Germany) (Figure 8E). ABCA1 down regulation was specific for group 1 – PAH, but not for CTEPH and COPD-PH (Figure 8F). Interestingly, we demonstrated that the ABCA1-activating compound T0901317 ameliorated MCT-induced PH with improvement in RV hypertrophy (Figure 9A), and pulmonary hemodynamics (decrease in mean pulmonary artery pressure (mPAP) and total pulmonary vascular resistance (TPR)) (Figure 9B and 9D), without affecting systemic pressures measured in carotid artery (CASP) (Figure 9E). The decrease in TPR in T0901317–treated PH rats was linked to a decrease in the percentage of the resistive fully muscularized distal microvessels (Figure 9H). Thus, these results suggest a role of ABCA1 in the pathophysiology of PAH.

**Table 5:** Gene expression meta-analysis using NextBio platform on all ABC transporters genes in PAH

ABC subtypes	Fold change in the bioset:” Lung idiopathic PAH versus healthy control” (Rajkumar R et al., 2010)
Abca1	−3.01
Abca8	−1.85
Abca9	−1.85
Abca10	−2.24
Abca11	−1.44
Abcb6	−1.87
Abcb10	−1.85
Acb12	−1.85
Abcc5	−2.38
Abcc6	1.86
Abcc9	−1
Abcd3	−2.5
Abcf2	−1.47
Abcg1	1.97

## DISCUSSION

**Figure 10:**
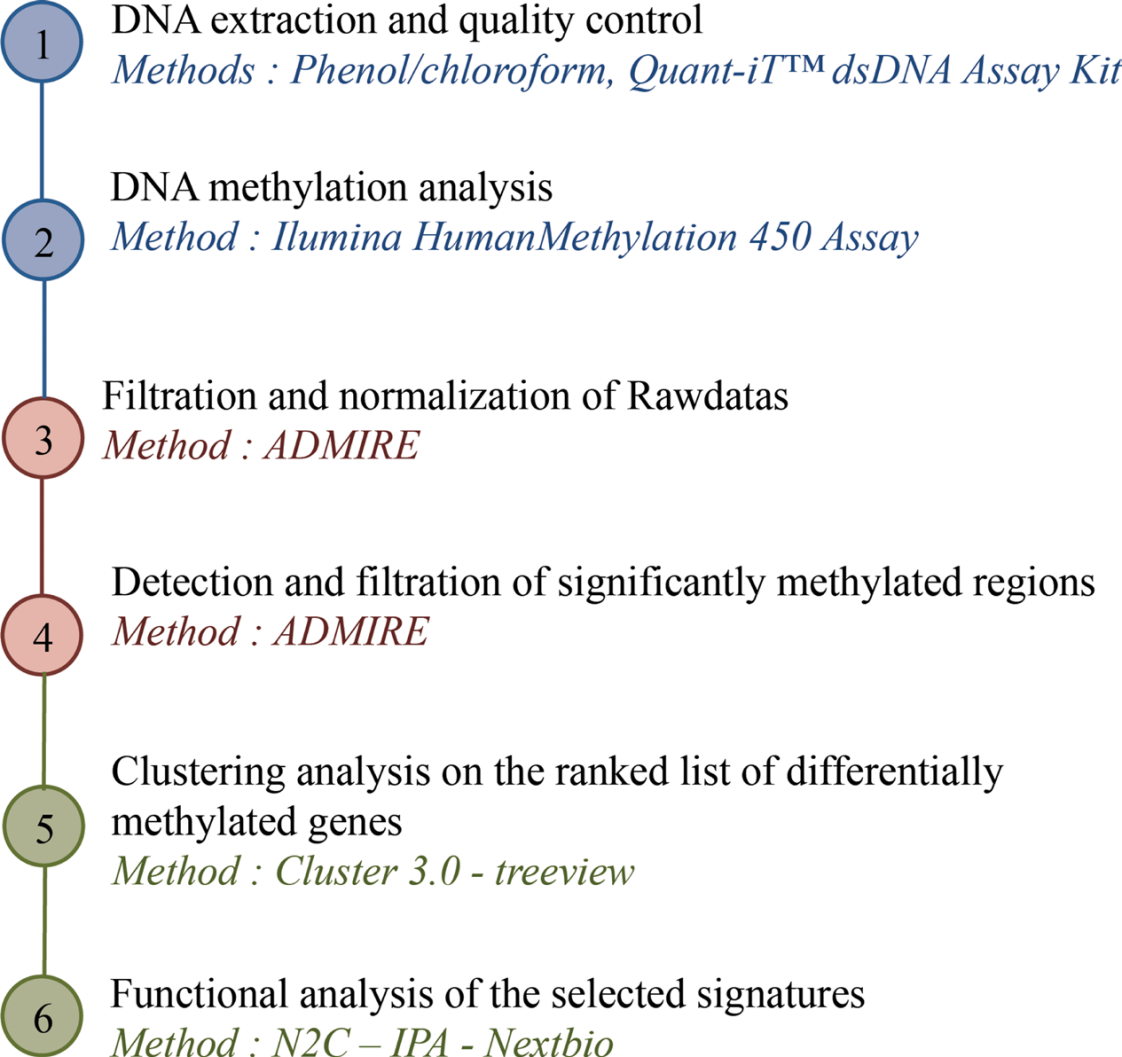
Strategy of DNA methylation analysis: from DNA to targeted promoters

We have addressed the inherited non mutation-associated causes of PEC proliferation in PAH. Indeed, a majority of PAH patients have no identified genetic triggers and PEC isolated from PAH lungs maintain their *in vivo* characteristics when placed in culture, suggesting epigenetic abnormalities [16]. Among epigenetic marks, we chose to analyze whole genome DNA methylation by combination of Infinium HumanMethylation450 assays and bioinformatic tools (Figure 10).

Cell culture has allowed substantial contributions to PAH translational research and biomedical discovery and are being widely used. Indeed, the conservation of an abnormal cellular phenotype outside their natural habitats has been demonstrated for primary cultures at early passages. In severe PAH, cultured PECs exhibit hyperproliferative and apoptosis-resistant phenotypes contributing to the extensive pulmonary vascular remodeling observed in PAH, as well as proinflammatory phenotypes [17]. For these reasons, we chose to use cultured PECs in our study, even if Nestor et al [18] demonstrated that a rapid, global loss of 5-hydroxymethylcytosine (5 hmC), but not 5-methylcytosine (5 mC), occurs during adaptation of mammalian cells to culture. This epigenetic remodeling was paralleled by extensive gene expression changes in culture, linked to cell adhesion and extracellular matrix organization. Actually, these changes probably reflect adaptation to growth on a two-dimensional plastic surface [18].

The genome-wide DNA methylation profile of PEC from PAH patients provides three differential signatures composed by hyper- or hypo-methylated promoters when compared to control. We observed that these patterns were not dependent of the age, gender and genetic causes of PAH (Figure 1A). Indeed, we showed that only three promoters (ZBTB18, AP001468.58, AC139100.2) were differentially methylated between idiopathic and heritable PAH (Figure 2A). ZBTB18 (also known as TAZ1 or RP58) has never been implicated in PAH, but its role in mitochondria [19] and as a transcriptional repressor regulating ID family genes, important downstream targets of BMP/ TGFb signalling [20] may be relevant in the disease pathophysiology [21–23]. Our analysis highlighted a similar methylation profile between heritable and idiopathic PAH (Figure 2B) which is concordant with a previous study showing an identical gene expression pattern in PASMCs from idiopathic and heritable PAH [24].

To characterize sets of differentially methylated promoters, we performed a gene ontology and network analysis. The hyper and hypo-methylated promoters in PAH compared to controls were mainly associated with angiogenesis and vascular diseases (Figures 1B, 3A, Tables 2, 3, 4). We observed a dysfunction in the promoters DES, ETS2, TERT, MYH7, TBX1/5 playing a major role in heart contraction and morphogenesis [25–28] but also in promoters involved in inflammation and remodeling (growth factor and cytokines activities) such as TNFSF4, COL1A1 and FGF1 [29, 30]. These results are in agreement with the central implication of inflammatory mechanisms in PAH [4, 31]. Additionally, our DNA methylation pattern highlighted a network of dysregulated promoters involved in adipogenesis particularly lipid and cholesterol transport (Figure 3B–3E). Among them, we identified the 8 most differentially methylated promoters including ETS2, STAT5A, miR26A, BCL2L11, ADIPOQ, ABCA1, APOA4 and ABCB4. By using IGV, public mRNA-expression data from Nextbio, and IPA, we concluded that ABCA1 was a major hyper-methylated promoter or downregulated gene discriminating PAH and control (Figures 5, 6, Table 5).

ABCA1 belongs to the super-family ATP Binding cassette (ABC) transporters. ABC transporters are important for pulmonary homeostasis, with at least three cases of persistent PH of the newborn due to loss-of-function mutations in *ABCA3* [32–34]. To date, no study has reported a relationship between ABCA1 promoters and the pathogenesis of PAH. This protein catalyses the transfer of lipids from various tissues and cells to apolipoprotein A1 containing lipoproteins [35]. This reaction is the rate-limiting step in the biogenesis of high-density lipoprotein (HDL) particles and reverses cholesterol transport [35]. HDL levels are a strong, independent inverse predictor of cardiovascular diseases. The protective effects of HDL cholesterol are most likely multifactorial, including reverse cholesterol transport to the liver [36], antioxidant properties [37], anti-inflammatory properties [38], protection of the endothelium [39], anticoagulant effects [40], activation of the endothelial nitric oxide synthase [41], and enhancement of the half-life of prostacyclin [42]. Interestingly, Guay et al. have recently shown that a higher DNA methylation level in the *ABCA1* gene promoter locus was associated with lower HDL-C levels and a previous history of coronary artery disease in familial hypercholesterolemia [43]. The authors confirmed in a second independent study that DNA methylation levels at the *ABCA1* gene promoter locus are associated with aging and coronary artery disease in men [44]. Other epigenetic studies showed that aging and prenatal famine exposure are associated with DNA hyper-methylation at the *ABCA1* gene promoter locus [45, 46]. Overall, these results suggest that both *in utero* and postnatal environments might modulate the *ABCA1* epigenetic profile and trigger a long-term susceptibility to cardiovascular diseases [47–49] including PAH [50–52]. This hypothesis has to be tested in the future. Moreover, the higher methylation identified in the *ABCA1* gene promoter may be responsible for the lower circulating HDL content found in PAH. Zamanian et al. [53] showed that female patients with PAH were more likely to be insulin resistant defined by a TG/HDL-C ratio greater than 3.0. These patients had significant lower circulating HDL-C level, independently of their BMI that was not different from controls. It has also been reported a significant decrease in circulating levels of HDL cholesterol in PAH patients, which were associated with worse clinical outcomes [54]. Strikingly, patients with low HDL-C had higher levels of IL-17A, an inflammatory characteristic that we recently identified in PAH [55]. Cracowski et al. [56] were not able to confirm the prognostic value of HDL cholesterol in a multicentre prospective cohort study of patients with incident PAH. However, a recent study performed on a larger cohort demonstrated that HDL was an independent predictor of survival [57]. This is in accordance with a recent study concluding that serum HDL cholesterol levels is a prognostic indicator in patients with idiopathic PAH [58].

Our bioinformatic analysis described a determinant role of the growth-suppressive and proapoptotic transcription factor TP53 in the regulation of the metabolic genes including ABCA1 (Figures 3 and 4). Using IPA, we showed that a predictive activation of *TP53* downregulates ABCA1. Such an activation is in contradiction with results obtained in experimental PH showing that at the whole-organism scale, inactivation of TP53 is sufficient to induce PH in rats [59] and that its activation prevents and reverses PH in mice exposed to chronic hypoxia or SU5416/hypoxia [60]. Inactivation of TP53 is expected to promote the aberrant cancer-like PASMC proliferation, responsible for medial hypertrophy and distal neomuscularization leading to pulmonary vasculature occlusion and increased pulmonary vascular resistance in these models of PH. However, at the specific endothelial level, activation of TP53 inhibits angiogenesis [61]. Hence endothelial specific TP53 activation may be responsible for the pulmonary vascular pruning, loss of cardiac and peripheral muscle microcirculation that increases pulmonary vascular resistance and contribute to exercise intolerance in human PAH [62, 63]. Herein, our methylation profile suggests that TP53 metabolic targets could play a role in impaired angiogenesis that is a neglected aspect of PAH pathobiology. However, even if TP53 is regulated mainly through protein turnover, its activity is also regulated through critical context-specific or fine-tuning events, mediated primarily through post-translational mechanisms, particularly multi-site phosphorylation and acetylation [64]. The regulation of TP53 in PAH endothelial cells remains a complex mechanism which deserves further consideration.

In summary, our methylation analysis provides the first map of DNA methylation-base epigenetic predispositions of PEC dysfunction in PAH. This approach allowed us to describe that PEC dysfunction in PAH is related to epigenetic modifications in genes involved in the cholesterol and lipid transport.

Our study has several strengths. The use of whole tissue results in the average of the various methylation profiles coming from diverse cell types. Additionally, lung sections may contain vessels of different sizes and may have differing cell composition, which makes it difficult to interpret the results with regard to relevant biological processes [65]. To overcome tissue heterogeneity and to specifically address the epigenetic origin of PEC dysfunction, we analyzed DNA from isolated primary PEC from controls and PAH patients. Moreover, mRNA-based expression profile may be lost during cell passage and directly influenced by culture conditions. We thus provided stable and long-lasting DNA-based methylation profile to identify non mutation-based epigenetic predispositions to PAH. Only two studies have analyzed the expression profile specifically in PEC from human and experimental PH [66, 67] and they only highlighted two well-known signaling pathways altered in PAH (BMPR2 downstream pathways and Notch signaling). However, both studies used very limited number of samples (three controls and PH cases). We performed a much more powerful study analyzing DNA from 11 idiopathic PAH, 10 heritable PAH, and from 18 controls. Importantly, we identified DNA methylated genes and gene networks that are commonly regulated between idiopathic and hereditary PAH. This can provide novel therapeutic options that can be useful for various subgroups of PAH. Our study has limitations as well. We focused on the methylation profile of gene promoters. Indeed, the methylation status of promoter associated CpG islands can directly affect gene transcription. However, the role of the CpG methylation outside the immediate promoter region remains somewhat unclear. Methylation of CpG island shores outside the promoter could also control transcription of downstream genes [68] or lead to histone modifications [69]. Methylation changes that occur in intragenic regions could impact RNA splicing [70]. In addition, methylation changes may affect the expression of non-coding RNAs [71] and thus indirectly affect global changes in gene expression. Another limitation of our methylation analysis is that our control tissues were from aged patients with lung cancer. The concept of field cancerization (accumulation of genetic and/or epigenetic alterations in normal-appearing tissues surrounding the cancer) has been indicated for liver, colon, Barrett's esophageal, lung, breast, and renal cancers, and can include changes in DNA methylation [72, 73]. Therefore, some differences between control and PAH could be related to cancer in the controls and not to PAH. However, the lung specimens from our controls were collected at distance from tumor foci. In any case to exclude the possibility of ABCA1 DNA methylation and subsequent gene regulation in the control tissues, we performed qPCR from the donor lung tissue (taken from the lung that was not transplanted). Indeed, we could confirm down regulation of ABCA1 expression in PAH tissues and endothelial cells compared to age- and sex- matched unused transplant donors (Figure 8A–8E), suggesting ABCA1 regulation is associated with PAH. Interestingly, there was no ABCA1 down regulation in CTEPH and COPD-PH lungs (Figure 8F), suggesting that ABCA1 is specifically downregulated in group 1 PH but not in at least group 3 and 4 PH. We didn't have tissues available from other PH groups. This qPCR-based confirmation of ABCA1 downregulation in PAH was carried over on lung tissues. It is therefore possible that ABCA1 downregulation affects other cell type of the pulmonary vasculature and parenchyma. The potential downregulation of ABCA1 in these other pulmonary cells like fibroblasts may also play a role in the PAH pathobiology [74]. Moreover, we confirmed the relevance of ABCA1 downregulation in PAH in an *in vivo* experiment. We designed an ABCA1 activation-based curative strategy for experimental PH induced in rat by monocrotaline (MCT) exposure. Since liver X receptor (LXR) regulates ABCA1 expression in endothelial cells [75], we treated MCT-exposed rats from day 14 to day 21 (symptomatic phase of the model) [76] with the LXR-activating ligand T0901317 at 10 mg/kg/day [77]. Importantly, the use of a ABCA1 activator (T0901317) improved MCT-induced PH at the hemodynamic and morphological levels (heart and pulmonary vasculature). This experiment validated ABCA1 downregulation as a pathomechanism of PAH and made the proof-of-concept that its targeting may offer novel therapeutic options.

In conclusion, unlike genetic mutations, epigenetic changes, and DNA methylation in particular, are pharmacologically reversible, which makes them an attractive target in future PAH drug development. Of note, two clinically used compounds with DNA demethylating activities, azacitidine and decitabine, have been recently approved for haematological malignancies [78]. Our present study has identified new pathological pathways potentially involved in PAH and proposes a new concept for PAH predisposition based on DNA methylation patterns.

## MATERIALS AND METHODS

### Patient groups

PAH patients studied in the methylation analysis were part of the French Network on Pulmonary Hypertension, a program approved by our institutional Ethics Committee, and had given written informed consent. Lung specimens were obtained during lung transplantation. Control patients had undergone lobectomy at our institution (Marie Lannelongue Hospital), either for adenocarcinoma or for squamous cell carcinoma and had given informed consent. Age was 34.4 ± 12.5 years and 36.4 ± 13.5 years in the patients with idiopathic PAH (IPAH) and heritable PAH (HPAH) due to *BMPR2* mutation (except for one case of familial PAH with no detectable mutation) and 60 ± 12.2 years in the control subjects. In the groups with IPAH and HPAH, the mean pulmonary artery pressure was, respectively, 61.1 ± 14.4 mmHg and 57.9 ± 9.9 mmHg; and mean pulmonary vascular resistance was 14.9 ± 6.9 and 9.4 ± 2 Wood units. Transthoracic echocardiography was performed preoperatively in the control subjects to rule out pulmonary hypertension.

mRNA and protein expression of selected targets were confirmed in totally unrelated pulmonary samples from University Hospital Giessen (Giessen, Germany). To rule out the possibility that some differences between control and PAH could be related to cancer in the controls and not to PAH, we used age- and sex-matched unused transplant donor lungs as controls in this validation analysis (mean age 52.7 ± 17.2 years, *n* = 7).

The study protocol for tissue donation was approved by the ethics committee (Ethik Kommission am Fachbereich Humanmedizin der Justus Liebig Universität Giessen) of the University Hospital Giessen (Giessen, Germany) in accordance with national law and with Good Clinical Practice/International Conference on Harmonisation guidelines. Written informed consent was obtained from each individual patient or the patient's next of kin (AZ 31/93). Mean Age was 43.8 ± 10.1 years in the patients with idiopathic PAH (IPAH) (*n* = 7), 55.1 ± 6.2 years in the patients with COPD associated PH (COPD-PH) (*n* = 9), 44.7 ± 5.8 years in the patients with chronic thromboembolic PH (CTEPH) (*n* = 8), and 52.7 ± 17.2 years in the control subjects (*n* = 7).

### DNA isolation and DNA methylation data analysis

PEC from patients with idiopathic PAH (*n* = 11), heritable PAH (*n* = 10) and from controls (*n* = 18) were isolated, then cultured as previously described [76] and were used at passage 3. Genomic DNA was extracted from cultured PEC by using the phenol/chloroform method including an RNase treatment. Extracted DNA were quantified for each sample with the kit Quant-IT (Quant-iT™ dsDNA Assay Kit, Thermo Fisher) and were controlled for quality by gel electrophoresis. DNA methylation was assessed at over 485,000 CpG sites using the Illumina Infinium^®^ HumanMethylation450 BeadChip. The slides were scanned by iScan+ (Illumina^®^, San Diego, USA). Raw data files were used as input for ADMIRE [79], a method for analyzing DNA methylation in genomic regions. Briefly, data was quality checked and normalized using the functional normalization developed by Aryee et al. [80]. Two one-sided two sample Mann-Whitney *U*-tests were done for each CpG site (post-filter) to account for non-normality of the data and to enable the combination of obtained *p*-values into genomic regions. CpG sites were mapped onto all 2 kb regulatory regions upstream a TSS annotated in GENCODE V19 [81] and comb-p [82] was used to obtain a multiple-testing corrected q-value for each regulatory region. A q-value of 0.05 (FDR) was used to filter significant differentially methylated regions. Following this method, three files were generated by comparison of the different groups (control versus all (heritable and idiopathic); control versus idiopathic; and control versus heritable) and containing normalized methylation values from differentially methylated regions. For each pairwise comparison, unsupervised hierarchical clustering was performed with Cluster (v3.0) using Spearman Rank correlation with a median-centered gene dataset. TreeView was used to generate the heatmaps and to select individual clusters containing hypermetylated or hypomethylated promoters.

Pearson correlation coefficients were calculated based on normalised methylation values using the R function cor from the base package. Briefly, the matrix of methylation values with rows corresponding to CpG sites and columns corresponding to samples was limited to CpG sites of interest (all promoters) as well as samples from iPAH or hPAH. Next, the correlation was calculated between the group-wise means of CpG site methylation values.

The methylation data reported in this paper have beens deposited in NCBIs Gene Expression Omnibus (GEO) (Omnibus, 2014) and are accessible with the access number (GSE84395).

### Functional and network analysis

Enrichment analysis of the protein coding-genes for each cluster was assessed using the tool (N2C) [83]. Given the genes of interest, N2C produces visual patterns on canvases of enriched terms. Each square on the canvas represents a functional term, and terms are organized on the canvas based on their gene-set content similarity. This method allows us to create canvases for the following gene-set libraries: GO ontologies (Biological process and molecular function), Wiki pathway and OMIM diseases. The enrichment results for each canvas were displayed in tables thanks to the Enrichr application (http://www.ncbi.nlm.nih.gov/pubmed/23586463) with only the most significant term (*p*-value < 0.05). In addition to N2C, we used Ingenuity Pathway Analysis (IPA) to confirm the functional annotations and to create biological networks. A fold change (FC) was attributed for each gene in function of the methylation or expression levels (positive FC = hypo-methylated or under-expressed genes (green); Negative FC = hyper-methylated or over-expressed genes (red)). In some cases, we extend the method to predict downstream and upstream effects on biological functions of regulated genes.

### Integrative genomic viewer (IGV)

To visualize the methylation region, we used the IGV tool that enables real-time exploration of large datasets including methylation [84]. All data files submitted to IGV are represented in horizontal rows called track. Figure 3D–3E display various tracks including the Genome reference Hg19 (RefSeq Genes), all human promoters (gene promoters), all existing CG probes (Illumina CG probes) together with all differentially methylated probes between control and PAH patients (Control vs all). Moreover, additional files from the heatmap analysis were loaded showing the hypo-methylated (cluster 1, Figure 3D) or hyper-methylated (cluster 2, Figure 3E) promoters in PAH patients compared to controls. By evaluating the methylation level (numbers of modified probes) for each promoter, IGV allowed us to focus on the most important genes.

### Meta-Analysis using the nextbio platform

To investigate the expression of gene of interest in PAH disease, the Nextbio application “disease atlas” was used (www.nextbio.com). Given a set of genes, Nextbio can explore gene functions and known associations with disease across more than 10,000 genomic studies contained in its library. Figure 5 shows the gene score based on statistical significance across the biosets (100 to the most significant inside the biosets). For each gene, Nextbio includes an activity value (or Fold change) represented in green (under-expressed gene) and red (overexpressed gene).

### Quantitative real time PCR (qPCR)

Total mRNA was extracted from frozen human lung tissues with the Trizol. Equal amounts of isolated RNA were subsequently transcribed into cDNA using the High capacity RNA-to-cDNA Kit (Applied Biosystems, Germany) according to the manufacturer's instructions. qPCR was then performed with the Taqman gene expression assay kit for ABCA1 and beta-actin (Applied Biosystems, Germany). Expression was analyzed with the ΔCt method. The Ct values of the target genes were normalized to that of the housekeeping gene (endogenous control) encoding beta-actin using the equation ΔCt = Ct_reference_ – Ct_target_ and expressed as ΔCt.

### Immunohistochemical detection of ABCA1 in paraffin-embedded lung tissues

The staining was performed with a VENTANA BenchMark ULTRA fully automated staining instrument (Roche, Schweiz). After classical dewaxing and heat antigen retrieval at pH 6 (citrate buffer, Protocol CC2), immunohistochemistry was performed with a mouse anti–ABCA1 (Thermo Scientific, France; Clone: HJ1, Dilution: 1/400, Incubation time: 32 min), detected by a biotinylated goat anti-mouse and streptavidin peroxidase (Thermo-Scientific, France) and permanent AEC kit (Ref ZUC054-200, Zytomed, Germany). An unrelated antibody of the same isotype as the primary antibody was used in negative controls. Slides were counterstained with hematein.

### *In vivo* study design, measurements, and tissue sampling

Experiments were conducted according to the European Union regulations (Directive 86/609 EEC) for animal experiments and complied with our institution's guidelines for animal care and handling. The animal facility is licensed by the French Ministry of Agriculture (agreement No. B92–019–01). The Committee on the Ethics of Animal Experiments CEEA26 CAPSud approved the study. Dr. Perros supervised all animal experiments (agreement delivered by the French Ministry of Agriculture for animal experiment No. A92–392). All efforts were made to minimize animal suffering.

Male Wistar rats (100 g body weight) were maintained in a temperature-controlled room with a 12/12-h light/dark cycle and randomly divided into: 1) a monocrotaline (MCT) and solvent (ethanol)-exposed group (MCT, *n* = 8); 2) a MCT-exposed and 10 mg/kg/day (days 14–21) T0901317-treated group (MCT+ T0901317, *n* = 8).

All rats had access to standard rat chow and water ad libitum. For MCT administration, rats received a single subcutaneous injection of 60 mg/kg MCT (Sigma-Aldrich, Lyon, France), which was dissolved in 1 N HCl and neutralized with 1 N NaOH. At day 21, measurement of the right ventricular systolic pressure (RVSP) (mmHg), mean pulmonary artery pressure (mPAP) (mmHg), mean carotid artery systemic pressure (mCASP) (mmHg), cardiac output (CO) (ml/min), and total pulmonary vascular resistances (TPR) (mmHg.ml^−1^.min) were recorded as previously described [85]. After rats were exsanguinated, the right lungs were distended by infusion of optimal cutting temperature compound (Miles, Epernon, France) diluted in phosphate-buffered saline (1:1) into the right principal bronchus, quick-frozen in isopentane on dry ice, and stored at −80°C. Left lungs were formalin- distended, fixed, and paraffin-embedded. For Fulton's index of right ventricular hypertrophy, the ratio of the right ventricular weight–to–left ventricular plus septal weight (RV/LV+S) was calculated.

## Abbreviations

ABC transporters: ATP Binding cassette transporters
BMPR2: Bone morphogenetic protein receptor type II
GEO: Gene Expression Omnibus
HDL: high-density lipoprotein
IPA: Ingenuity pathway analysis
PAH: Pulmonary arterial hypertension
PASMC: pulmonary arterial smooth muscle cells
PEC: pulmonary endothelial cells
